# Acute Responses of Cardiac Biomarkers to Intermittent and Continuous Exercise Are Related to Age Difference but Not I/D Polymorphism in the ACE Gene

**DOI:** 10.3389/fphys.2020.00665

**Published:** 2020-07-02

**Authors:** Akram Falahati, Hamid Arazi, Katsuhiko Suzuki

**Affiliations:** ^1^Department of Exercise Physiology, University of Guilan, Rasht, Iran; ^2^Department of Exercise Physiology, Faculty of Sport Sciences, University of Guilan, Rasht, Iran; ^3^Faculty of Sport Sciences, Waseda University, Tokorozawa, Japan

**Keywords:** exercise, cTnI, NT-proBNP, middle-aged, young, ACE gene

## Abstract

**Purpose:**

The purpose of this study was to determine the (i) cardiac biomarker (cTnI and NT-proBNP) responses to moderate-intensity continuous exercise (MICE) and high-intensity interval exercise (HIIE) in the middle-aged and young groups, (ii) relationship of post-exercise cardiac biomarker release between these two age groups, and (iii) investigate whether insertion/deletion (I/D) polymorphism in the angiotensin-converting enzyme (ACE) gene is associated with predisposition to cardiac damage after exercise in Iranian men.

**Methods:**

We examined cTnI and NT-proBNP in 29 middle-aged (54.5 ± 4.6 years) and 28 young (22.7 ± 4.2 years) soccer players before and after HIIE and MICE running tests.

**Results:**

The middle-aged soccer players had higher baseline cTnI (0.015 ± 0.007 ng/ml vs. 0.010 ± 0.006 ng/ml; *P* = 0.01) and NT-proBNP (30.7 ± 13.6 ng/L vs. 18.4 ± 8.3 ng/L; *P* < 0.001) values compared with the young group. The changes with exercise (ΔcTnI: 13 vs. 11 ng/ml and ΔNT-proBNP: 18 vs. 11 ng/L) were also higher in the middle-aged group. No subject exceeded the upper reference limit for cTnI and NT-proBNP. Considering three ACE genotypes, the mean cTnI and NT-proBNP values of middle-aged and young groups did not show any significant difference.

**Conclusion:**

Marked differences in baseline and post-exercise cTnI and NT-proBNP values were observed, which were related to age differences but not to ACE genotypes.

## Introduction

The health-promoting and preventive potential of regular physical exercise to reduce cardiovascular disease and the risk of premature death is undeniable, although some people, mainly those who are not adapted to training, experience exercise-related sudden cardiac arrests (SCA). Middle-aged adults (35–59 years) make up a large part of the SCA cases (30–40%) (Noheria et al., 2013), which is a public health concern. The growing popularity of exercise in an aging population urges the necessity to understand the physiological changes that occur with aging, when new approaches to prescribing an exercise mode are to be adopted.

In comparison with traditional moderate-intensity continuous exercise (MICE), high-intensity interval exercise (HIIE) has been promoted as an effective mode of exercise which improves cardiovascular and metabolic function (Gibala et al., 2012) and even alters the quality of life at advanced ages (Winett and Ogletree, 2019). Regardless of the described benefits of aerobic HIIE, concerns have been raised about the safety and the direct effects of HIIE on the heart (Li et al., 2020) because the aged heart is exposed to increasing levels of stress due to its decreased functional reserve. As a result, the induced high cardiorespiratory load of HIIE may elicit a prominent unpleasant increase in cardiac biomarkers which are indicative of cardiac damage (Strait and Lakatta, 2012).

In the cardiovascular setting, it is preferred to use different marker tests in varying combinations, such as combining troponin (cTn), a marker for cardiac injury, and N-terminal pro-brain natriuretic peptide (NT-proBNP), a biomarker that does not come from myocardial necrosis but by different mechanisms such as myocardial stretch, inflammation, or stress (Rains et al., 2014).

In studying the effects of age on cardiac response to exercise, it is important not to consider the heart as an isolated organ since it is in series with the vascular system. The angiotensin-converting enzyme (ACE) gene is the candidate gene involved in cardiac growth and function. The endocrine renin–angiotensin system (RAS) plays a fundamental role in controlling the circulatory system. Angiotensin II (Ang II), a major effector of RAS, is involved in the homeostasis of fluid *via* its effects on aldosterone secretion and is a powerful vasoconstrictor, resulting in saltwater retention and endothelial dysfunction, which is a major public health concern.

The mitogen-activated protein kinase, phosphoinositide-3-kinase/AKT, and protein kinase cAMP-dependent pathways are among the most ancient signal transduction pathways activated by angiotensin II that play a central role in the control of cell proliferation, growth, and differentiation (Mehta and Griendling, 2007). The presence or absence of the 287 bp Alu sequence in intron 16 of the ACE gene forms the insertion (I allele) and/or deletion (D allele) polymorphisms, which contribute to three genotypes – insertion homozygote (I/I), insertion/deletion heterozygote (I/D), and deletion homozygote (D/D). The D allele is associated with peak ACE levels in the circulatory system and the ventricular tissue (Raynolds et al., 1993). Several studies have shown the association of D allele and DD genotype with heart failure, systolic dysfunction, and acute myocardial infarction (Mendonça et al., 2004; De Boer et al., 2009; Zhou et al., 2012; Hmimech et al., 2017). Therefore, a positive association between the D allele and elevation of exercise-induced cardiac biomarkers indicative of cardiac damage is suggested. However, the amount of this release may be importantly affected by the age of the subjects because aging results in a lower injury threshold for cardiac damage (Noheria et al., 2013). In addition, the transcription, translation, and activity of ACE markedly increase within the arterial wall with aging (Wang et al., 2003). As a result, Ang II protein becomes markedly increased, particularly in the thickened intima. The Ang II receptor, AT1, is also upregulated within the old arterial wall (Wang et al., 2007).

To have a preventive approach, the health benefits of studying on middle-aged group are enormous because the majority of the Iranian elderly population currently suffer from a variety of chronic diseases, with cardiovascular disease and reduced cardiac function being especially prevalent. Thus, the present study aimed to examine the possible risk outcomes of HIIE on the middle-aged (<60) rather than the older population. The current study hypothesized that HIIE may elicit a much higher increase in cardiac biomarkers indicative of cardiac damage compared with MICE, and this would be more apparent in the middle-aged compared with the young group. A positive association between the D allele and the DD genotype with an elevation in exercise-induced cardiac biomarkers indicative of cardiac damage is also suggested. The present study may be summarized to the following three parts: (part I) cardiac biomarkers (NT-proBNP and cTnI) responses to acute intermittent and continuous exercise, (part II) relationships between post-exercise cardiac biomarker release between the middle-aged and the young groups, and (part III) the influence of the ACE gene I/D polymorphism on cardiac biomarker release with specific regard on the influence of the age category.

## Materials and Methods

### Design Overview

A pre- and post-test design was employed in order to measure the effects of MICE and HIIE on specific cardiac biomarkers in middle-aged and young male subjects.

### Participants

Soccer players (white men of Iranian/Persian descent) were enrolled *via* an open invitation from a big soccer club. The subjects included all athletes participating in soccer at a competitive or masters level (*n* = 29), who had at least a history of 5 years of regular soccer training or leisure activity subjects (*n* = 28), and who were active members of the soccer community with no regular training program. In total, 57 individuals were included in the genotyping phase of the study. The ACE-I/D polymorphism was conducted in a manner that was blinded to the clinical outcomes of cardiac biomarkers. For the analysis, the subjects were categorized based on their age group and ACE I/D genotype as assessed by a questionnaire and genotyping. The volunteers included 29 middle-aged (aged 45–60 years) and 28 young (aged 18–30 years) soccer players. The middle-aged subjects did not differ from the young group in their ethnic and geographical distribution throughout Iran. All the subjects were free from any renal, musculoskeletal, metabolic, and cardiorespiratory disorders. All the participants were asked to refrain from strenuous exercise as well as smoking and ingesting alcohol and caffeine for at least 48 h prior to the test session.

### Ethics Statement

This study was conducted in accordance with the Declaration of Helsinki and was approved by the institutional research committee of the University of Guilan (Ref. DT/17012017). The confidentiality of the participants was strictly maintained. After a full explanation of the study, the participants signed a voluntary written informed consent.

### Anthropometric and Physical Performance Measurement

At the first stage, we evaluated clinical assessments including anthropometric measurements. Height, weight, and skinfold measurement were taken in duplicate, and the average of the two was recorded. Self-determination of resting heart rate (RHR) for three mornings before getting up from bed was conducted by the subjects, following the instructions of the researcher. Blood pressure was measured before the exercise, in sitting position with the arm supported so that the elbow was at about the heart’s level, using an automated brachial oscillometric device (M10-IT; Omron, Kyoto, Japan). The calculation of VO_2__max_ of each soccer player was determined using Bruce protocol on a treadmill (HP Cosmos, Germany), in accordance with the recommended standards of the American College of Sports Medicine. The final result of the Bruce test is exercise time (ET) by which VO_2__m__ax_ can be accurately estimated (Pollock, 1977).

On the second and third visits and in a randomized crossover design which was separated by 7 days, the participants underwent two running exercise protocols of HIIE and MICE, which were adjusted to attain similar total work (i.e., isocaloric). The testing sessions were conducted during a preparatory period: 2–3 h after a standardized breakfast in an air-conditioned laboratory, with the temperature and the relative humidity specified as 20°C and 50%, respectively. Both running protocols were performed at the same time of the day to ignore circadian effects on the physiological characteristics (Reilly and Brooks, 1986) and on an identical motorized treadmill (HP Cosmos, Germany). The HIIE running protocol opened with a 7 min warm-up at 70% VO_2__max_, followed by six 3 min bouts at 90% VO_2__max_. Between the intervals, 3 min active recovery periods at 50% VO_2__max_ were arranged. Lastly, the participants performed a 7 min cool-down at a running velocity corresponding to 70% VO_2__max_. Accordingly, the exercise protocol included 18 min of high-intensity exercise and 18 min of active recovery which, considering warm-up and cool-down, granted a total duration of 50 min. The average intensity during HIIE protocol was equalize to 70% VO_2__max_. The MICE protocol was made up of 50 min of continuous running at 70% VO_2__max_. So, the two exercise protocols were matched for exercise duration (50 min), average intensity (70% VO_2__max_), and distance run (9,843 + 494 m) (Bartlett et al., 2011). A heart rate monitor (Polar S610i, Kempele, Finland) was used to control the heart rate during exercise.

### Blood Sampling

Blood samples were obtained just prior (pre) and within 15 min after the workout (post). The subjects were seated for 10 min prior to each blood collection. The samples were collected in serum separation tubes, allowed to clot for 30 min, and then centrifuged at 2,000 *g* for 7 min; the serum was then drawn off and stored at −80°C for later analysis.

The serum NT-proBNP level was determined using the Human N-terminal Pro-brain Natriuretic Peptide ELISA Kit (Bioassay Technology Laboratory Co., China, catalog number: E1239Hu). The sensitivity of the test is 4.12 ng/L; the measuring range is 8–1,900 ng/L.

The cardiac troponin I (cTnI) concentration was measured using Access AccuTnI assay (Beckman Coulter Inc., Fullerton, CA, United States). The upper reference limit (URL) for cTnI, defined as the 99th percentile for the healthy participants, was 0.04 ng/ml (Eggers et al., 2007).

### Genotyping of the ACE Gene I/D Polymorphism

Genomic DNA extraction from whole-blood samples was conducted using the salting out procedure (Miller et al., 1998). We managed polymerase chain reaction (PCR) to genotype DNA samples. The sequences of the sense and the antisense primers were 5’-CTGGAGACCACTCCCATCCTTTCT-3’ and 5’-GATGTGGCCATCACATTCGTCAGAT-3’, respectively. The PCR program used was as follows: denaturation at 95°C for 1 min, annealing at 30 cycles of 95°C for 1 min, 58°C for 1 min, 72°C for 2 min, and extension at 72°C for 10 min. The PCR product was analyzed by electrophoresis at 80 V for 60 min using 1.5% agarose gel. In the blind experiments, the reliability of genotyping was evaluated. The reactions confirmed the presence of the D (190 bp) and I (490 bp) alleles which clarify three profiles: II (490 pb), ID (490 and 190 bp), and DD (190 pb). This research was implemented in the molecular laboratory of Guilan University, Iran.

### Statistical Analyses

Data are presented as mean ± SD. The allele and the genotype frequencies in middle-aged and young individuals were compared using the chi-square test (χ^2^-test). Two-way analysis of variance (factors: age and ACE I/D polymorphism) was used to assess mean differences. A confidence interval of 95% significance was approved at *P* ≤ 0.05). The data were analyzed using SPSS version 16.0 (Chicago, IL, United States).

## Results

### Characteristics of the Subjects in Relation to Age Group

Twenty-nine of the 57 studied subjects were classified as middle-aged; the other 28 subjects, being young, were all included in the genotyping phase of the study. Fifty-six participants were able to complete both MICE and HIIE sessions, while one middle-aged subject stopped the MICE session after 15 min of running and hence was excluded from the analysis. Table 1 summarizes the characteristics of the subjects in dependence of the age category. The middle-aged and the young men had similar height, whereas weight (78.0 vs. 73.2 kg), body mass index (BMI; 24.1 vs. 22.4 kg ⋅ m^–2^), body fat mass (17.2 vs. 14.7%), resting systolic blood pressure (129 vs. 124 mmHg), diastolic blood pressure (78 vs. 73 mmHg), and RHR (62 vs. 59 b. min ^–1^) were significantly (*P* < 0.01) higher in the middle-aged group, and as expected, ET (14.3 vs. 12.0 min) and VO_2__max_ (52.7 vs. 43.5 ml ⋅ kg^–1^ ⋅ min^–1^) were higher in the young group (Table 1).

**TABLE 1:** Baseline characteristics of middle-aged and young males.

**Variable(s)**	**Middle-aged (*n* = 28)**	**Young (*n* = 28)**	**Statistical significance**
Age (years)	54.54.6	22.74.2	*P* < 0.001*
Weight (kg)	78.07.5	73.26.3	*P* = 0.01*
Height (cm)	179.74.4	180.85.7	NS
BMI (kg⋅m^–2^)	24.11.9	22.41.2	*P* < 0.001*
Fat mass (%)	17.23.0	14.74.0	*P* = 0.01*
Exercise time (min)	12.02.3	14.32.5	*P* < 0.001*
VO_2max_ (ml⋅kg^–1^⋅min^–1^)	43.59.3	52.710.1	*P* < 0.001*
Resting SBP (mmHg)	1293	1244	*P* = 0.003*
Resting DBP (mmHg)	787	737	*P* < 0.001*
RHR (beats min ^–1^)	625	594	*P* = 0.007*
cTnI (ng/ml)	0.0150.007	0.0100.006	*P* = 0.018*
NT-proBNP (ng/L)	30.713.6	18.48.3	*P* < 0.001*

Values are means ± SD; *P < 0.05. BMI, body mass index; SBP, systolic blood pressure; DBP, diastolic blood pressure; RHR, resting heart rate; cTnI, cardiac troponin I; NT-proBNP, N-terminal pro-brain natriuretic peptide; NS, not significant.

### cTnI

The pre-exercise cTnI data were significantly higher in the middle-aged subjects (middle-aged: 0.015 ± 0.007 ng/ml; young: 0.010 ± 0.006 ng/ml; *P* = 0.018) (Table 1). All the participants presented with an increase in cTnI at post-exercise, with the URL for cTnI not exceeded by any subject (<0.04 ng/ml). The middle-aged group had a higher post-exercise cTnI release, which was largely explained by the higher baseline values. The increase of cTnI concentrations (ΔcTnI) was also higher in the middle-aged group compared with the young group (13 vs. 11 ng/ml). Interestingly, there was no significant difference between HIIE and MICE in the amount of cTnI released (Table 2). The serum concentration of cTnI was < 0.04 ng/ml in all the participants at baseline and did not exceed this value after the exercise sessions.

**TABLE 2:** cTnI and NT-proBNP after HIIIE and MICE in middle-aged and young males.

	**Middle-aged**			**Young**			**Interaction**		
**Variable(s)**	**Pre**	**HIIE**	**MICE**	**Pre**	**HIIE**	**MICE**	**Age status**	**Exercise mode**	**Interaction**
cTnI (ng/ml)	0.015 ± 0.007	0.028 ± 0.001	0.028 ± 0.001	0.010 ± 0.006	0.021 ± 0.002	0.021 ± 0.002	*P* < 0.05*	NS	*P* < 0.05*
△cTnI (ng/ml)	0.013 ± 0.006			0.011 ± 0.004			*P* < 0.05*	NS	*P* < 0.05*
NTproBNP (ng/L)	30.7 ± 13.6	47.3 ± 23.9	48.4 ± 30.2	18.4 ± 8.2	29.7 ± 12.1	29.6 ± 11.1	*P* < 0.01*	NS	*P* < 0.05*
△NTproBNP (ng/L)	18.3 ± 19.7			11.3 ± 3.6			*P* < 0.05*	NS	*P* < 0.05*

Values are means ± SD; *P < 0.05. cTnI, cardiac troponin I; NT-proBNP, N-terminal pro-brain natriuretic peptide; HIIIE, high-intensity interval exercise; MICE, moderate intensity continuous exercise; NS, not significant.

### NT-proBNP

There was a significant difference in the pre-exercise NT-proBNP values between the middle-aged and the young subjects (30.7 ± 13.6 ng/L and 18.4 ± 8.3 ng/L, respectively; *P* < 0.001) (Table 1). There was a rise in NT-proBNP at post-exercise in all subjects, but the URL was not exceeded by any subject. There was a significant main effect of age on NT-proBNP data; as a result, the increase of NT-proBNP concentrations (ΔNT-proBNP) was higher in the middle-aged group compared with the young group (18 vs. 11 ng/L). The concentration of NT-proBNP increased significantly after HIIE (middle-aged: 47.3 ± 23.9 ng/L; young: 29.7 ± 12.1 ng/L) and MICE (middle-aged: 48.4 ± 30.2 ng/L; young: 29.6 ± 11.1 ng/L) (*P* < 0.01). However, no significant variations were found in the NT-proBNP levels between both exercise modes (Table 2).

### ACE Gene Characteristics of the Subjects in Relation to Age

**FIGURE 1:**
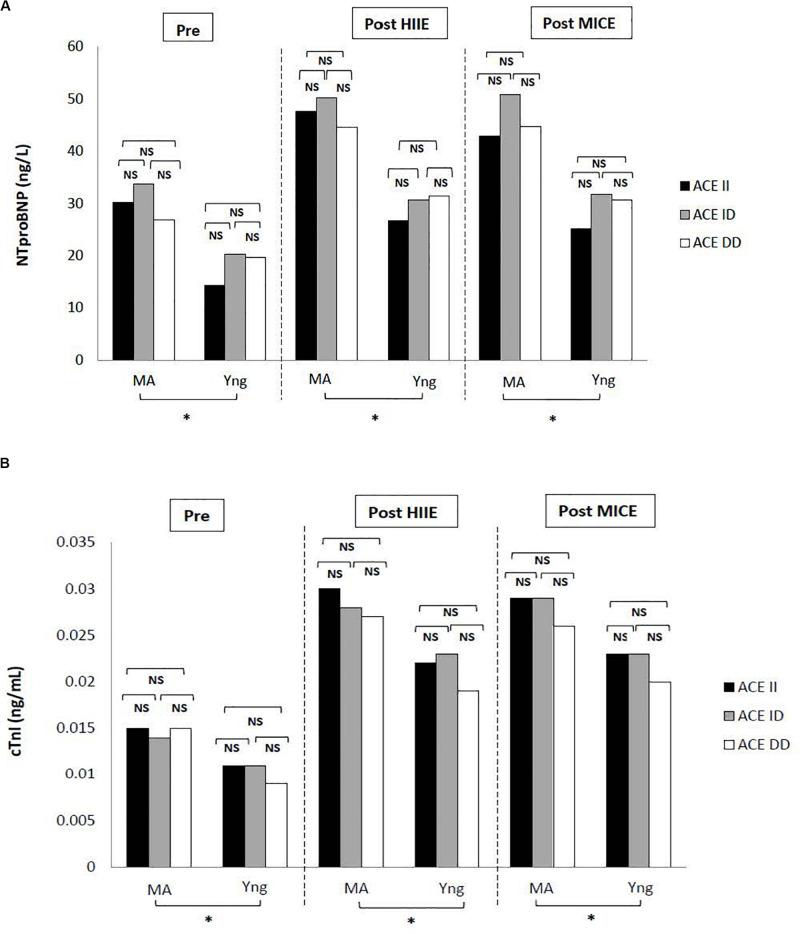
Pre- and post-exercise **(A)** NT-proBNP and **(B)** cTnI values of different ACE genotypes in middle-aged and young males. Data are shown as mean ± SD; ^∗^*P* < 0.05. NS, not significant; MA, middle-aged; Yng, young; cTnI, cardiac troponin I; NT-proBNP, N-terminal pro-brain natriuretic peptide.

The frequency distribution of ACE genotypes in the 56 Iranian males in the present study was as follows: middle aged: DD – 35.7%, *n* = 10; ID – 46.4%, *n* = 13, and II – 17.9%, *n* = 5; young: DD – 28.6%, *n* = 8, ID – 42.9%, *n* = 12, and II – 28.6%, *n* = 8. The frequency of the I and D allele was quite similar in the young group (50%, *n* = 28), but in the middle-aged group the frequency of the D allele (58.9%, *n* = 33) was significantly higher than the I allele (41.1%, *n* = 23) (Table 3). The distribution of the ACE genotype was in Hardy–Weinberg equilibrium. Considering three ACE genotypes, the mean pre-exercise values of cTnI in the middle-aged group (DD: 0.015 ± 0.008 ng/ml, ID: 0.014 ± 0.006 ng/ml, and II: 0.015 ± 0.009 ng/ml) and in the young group (DD: 0.009 ± 0.005 ng/ml, ID: 0.011 ± 0.007 ng/ml, and II: 0.011 ± 0.004 ng/ml) and NT-proBNP in the middle-aged group (DD: 26.9 ± 14.4 ng/L, ID: 33.7 ± 15.1 ng/L, and II: 30.2 ± 5.0 ng/L) and in the young group (DD: 19.6 ± 11.6 ng/L, ID: 20.3 ± 7.4 ng/L, and II: 14.3 ± 3.9 ng/L) did not show any significant difference (Figure 1). No interaction effect of age × ACE genotype was found for each of the biomarkers examined (Figure 1).

**TABLE 3:** Distribution of angiotensin-converting enzyme I/D polymorphism genotypes and allele frequencies between middle-aged and young males.

**Genotype**	**Middle-aged (*N* = 28)**		**Young (*N* = 28)**		***P*-value**	**OR (95% CI)**
	**Count**	**Frequency (%)**	**Count**	**Frequency (%)**		
DD	10	35.7	8	28.6	0.7	0.2
ID	13	46.4	12	42.9		
II	5	17.9	8	28.6		
D	33	58.9	28	50	0.7	0.5
I	23	41.1	28	50		

**II/ID/DD, ACE genotypes; I, insertion; D, deletion.**

## Discussion

In comparing HIIE and MICE, we found that the post-exercise increases of cTnI were comparable. In some studies, endurance exercise (Benda et al., 2015) and, in some others, a higher exercise intensity (Serrano-Ostáriz et al., 2011; Eijsvogels et al., 2014; Li et al., 2020) led to a larger cardiac biomarker release, indicative of acute cardiac damage. Our results in middle-aged and young soccer players extend the findings of Normandin et al. (2013), who indicated that cardiopulmonary responses did not differ between MICE and HIIE in patients (aged 61 ± 9.9 years) with heart failure (Normandin et al., 2013).

Aging may be one of the factors that determine the heterogeneity in baseline cTnI. The effects of aging are most evident in an exercise with a ΔcTnI value (13 vs. 11 ng/ml) higher in the middle-aged group compared with the young group. Being of middle age is a known cardiovascular risk factor. Middle-aged runners may experience higher loads to their cardiac muscle when they conduct a higher-intensity exercise than usual (Kim et al., 2015). The greater pre- and post-exercise cTnI levels in our middle-aged group are consistent with a study which has linked greater exercise-induced levels of cTnI to higher age (Eijsvogels et al., 2010) but contrary to some other studies which have associated it with a younger age (Fortescue et al., 2007; Benda et al., 2015). In the study of Eijsvogels et al. (2010), cTnI was increased above the clinical cutoff value for myocardial infarction, but in our study the cTnI levels were well below the cutoff point. The contradictions with previous studies may be related to the different training status or exercise protocols (Legaz-Arrese et al., 2015) or because, in those studies, the subjects with asymptomatic cardiac structural disease, minor ECG abnormalities, and hypertension were not excluded. Webb et al. (2015) confirmed that the relationship between age and hs-TnT was independent of co-morbidities (Webb et al., 2015). The possible reason for the higher cTn levels in older participants is because they were older and ran at relatively higher exercise intensity. An overall decrease in exercise tolerance is evident in the exercise time (12.0 vs. 14.3) and, as a result, in VO_2__max_ (43.5 vs. 52.7) which is lower in the middle-aged group. Exercise leads to a rise in stroke volume and heart rate in order to increase cardiac output. The constant increase in cardiac work may cause expanded mechanical stress on the heart which, in combination with the physiologic setting of prolonged exercise (e.g., elevations in reactive oxygen species, altered pH, and increased core temperature) could damage cardiomyocytes (Shave et al., 2010). The limited post-exercise amounts of cardiac damage are later repaired due to the normal regenerative capacity of the myocardium, which could be associated to a remodeling process (Middleton et al., 2008). Another explanation may be reduced renal clearance, with the mean estimated creatinine clearance in normal older subjects significantly lower than that in normal younger subjects (74 vs. 91 ml/min/1.73 m^2^, respectively, *P* < 0.0001) (Galasko et al., 2005), possibly resulting in a small increase in cTnI concentration in the blood. A meta-analysis (Shave et al., 2007) confirmed that the mild release of cTnI (range 0.02–0.04 ng/ml) begins after only 30 min of exercise and is a normal or “physiologic” response to exercise.

When the cTnI values are high, NT-proBNP determination has been proven to be a useful value in excluding the need for more cardiac interventions (Scharhag et al., 2004). We found that HIIE and MICE lead to equal NT-proBNP values indicative of myocardial wall stress. It was indicated that a shorter but more intense exercise also results in an increase of NT-proBNP among healthy individuals (Scharhag et al., 2008). However, this increase is shorter in time and does not exceed the URL. Previous endurance exercise studies (Neilan et al., 2006; Serrano-Ostáriz et al., 2009) revealed a higher NT-proBNP value compared with our study, which is expected as BNP is elevated in response to volume overload and myocyte stretch (Shave et al., 2007), and these stressors are more likely to occur during an endurance exercise (Scharhag et al., 2008).

In the present study, the pre- and post-exercise concentrations of NT-proBNP were significantly higher in the middle-aged group compared with the young group, but the values were similar to those reported for normal healthy individuals and did not exceed the URL. Furthermore, our pre-exercise results in the middle-aged group agree with the study of Galasko et al. (2005), who found the following cutoff value: 100 pg/ml for men aged 45–59 (Galasko et al., 2005). Increases in BNP and NT-proBNP have been related to the athlete’s age (Scharhag et al., 2005), and higher URLs in older subjects (Raymond et al., 2003) and after exercise have to be considered. The reason for the higher NT-proBNP values in the middle-aged group compared with the young group is not completely understood. The negative association between obesity and NT-proBNP level has been strongly confirmed (Yamashita et al., 2014). Noheria et al. (2013) showed a significant and independent association in middle-aged cardiac patients with obesity. Hookana et al. (2011) confirmed that the prevalence of cardiomyopathy related to obesity in the middle-aged group (between 40 and 59 years) was 23.7% and even higher than that in older than 60 years (22.8%) (Hookana et al., 2011). Our middle-aged group is not obese, but the higher BMI and fat mass compared with the young group (BMI: 24.1 vs. 22.4 kg⋅m^–2^; fat percentage: 17.2 vs. 14.7%) may have contributed to the higher NT-proBNP values. The probable mechanisms for cardiac damage due to fat accumulation include ventricular remodeling (left ventricular systolic and diastolic dysfunction), pro-arrhythmic epicardial fat, and accelerated coronary atherosclerosis (Hookana et al., 2011).

The central role of RAS has been suggested for age-related cardiac remodeling. As the pre- and post-exercise cTnI and NT-proBNP values were different between the middle-aged and the young subjects, the current study’s aim was to determine if these differences were related to particular genotypes of the ACE. In the Iranian population, the association of the D allele with cardiovascular dysfunction (Firouzabadi et al., 2012; Bahramali et al., 2016) has been previously established. At baseline, it was observed that the ACE gene variant genotypes were not different between the two age groups. The I and D alleles in the young group were equal (50%), but the frequency of the D allele in our middle-aged group (58.9%) was higher than the I allele (41.1%), which may have been the reason for the higher cTnI and NT-proBNP in this group. However, we did not find any associations between the I/D polymorphism and the cTnI and NT-proBNP values in an univariate analysis, which is in accordance with a previous study on 241 cardiac patients (Alazhary et al., 2015), and a meta-analysis (Bai et al., 2012) on the association of ACE gene with heart disease. Besides that, in a recently published study by the author (Falahati and Arazi, 2019), I/D genotype was associated neither with VO_2__max_ nor with RHR and blood pressure. In contrast, in some other studies, patients with the DD genotype compared with the ID/II groups had higher (*P* < 0.05) NT-proBNP (Palmer et al., 2003) and a greater risk of coronary artery disease (Amara et al., 2018). The underlying reasons for the observed disagreements may be ethnic as well as geographic differences (Nakhjavani et al., 2007), using individuals with mixed heritage and none of Asian ethnicity (Albuquerque et al., 2014), choosing cardiac patients (Palmer et al., 2003), and age and sex difference. These are potential epigenetic modifiers that can influence gene expression, molecular function, and probably the response of cardiac biomarkers (Paul et al., 2015). Thus, it is obvious that more studies are needed to completely comprehend the function of the ACE gene and the possible effects of its DNA sequence variation on cardiac response in different age groups. While our pre- and post-exercise cTnI and NT-proBNP values were generally different in the middle-aged and the young subjects, we found no evidence of genetic association in the phenotypes associated with the ACE I/D polymorphisms to the difference in cardiac biomarkers in this study.

### Strengths and Limitations

The strengths of this study include the examination of an interaction effect of age and ACE genotype on two important cardiac biomarkers (hs-cTnI and NT-proBNP), following two matched exercise protocols (HIIE and MICE), and the confirmation that age (but not the D allele of ACE gene) has no effect on pre- and post-exercise biomarker release.

There are some potential limitations that deserve to be mentioned. Because of the small sample size, the results should be clarified with caution and confirmed in a larger study population. Besides that, our study was constricted by limited blood sampling (pre- and post-exercise design). A major limitation is that the participants in this article are soccer players at different training levels (competitive or leisure activity). Finally, in addition to acute cardiac biomarker response to exercise, the possible cardiac adaptation due to chronic interval and continuous training was not conducted in a long-term clinical follow-up in this study. This paper examines the interaction effect of age and ACE genotype on cTnI and NT-proBNP, following HIIE and MICE, and confirms that age (but not the D allele of ACE gene) has no effect on pre- and post-exercise biomarker release.

## Conclusion

In conclusion, our results show that HIIE and MICE results in the elevation of both cTnI and NT-proBNP in all subjects, with no cTnI or NT-proBNP values above the URL. HIIE elicits similar cardiac biomarker responses as MICE in middle-aged and in young subjects. There is increasing recognition that this mild increase in serum cTnI and NT-proBNP, following exercise, is not associated with symptoms of cardiac injury but relates to a physiological response to exercise. The pre-exercise values of cTnI and NT-proBNP were highly variable, and there seems to be an important role for age difference in mediating the pre-exercise differences in middle-aged and in young subjects. Finally, the distribution of the D allele of the ACE gene was higher in middle-aged subjects compared with the young subjects, but with no interaction effect on pre- and post-exercise biomarker release.

## Data Availability Statement

The datasets generated for this study are available on request to the corresponding author.

## Ethics Statement

The studies involving human participants were reviewed and approved by the University of Guilan. The patients/participants provided their written informed consent to participate in this study.

## Author Contributions

HA made substantial contributions to the conception and design of the research. HA and AF had fundamental roles in the acquisition of data, analysis, and interpretation of data. KS made a substantial contribution in the conception and critical revision of the article. All authors were involved in writing the manuscript, had given final approval of the submitted version, and read and approved the final manuscript.

## Conflict of Interest

The authors declare that the research was conducted in the absence of any commercial or financial relationships that could be construed as a potential conflict of interest.
